# High Glucose Sensitizes Male and Female Rat Cardiomyocytes to Wnt/β-Catenin Signaling

**DOI:** 10.3390/biom14121639

**Published:** 2024-12-20

**Authors:** Ruonan Gu, Jerry Wang, Julianne Morin, Aizhu Lu, Wenbin Liang

**Affiliations:** 1 Cardiac Electrophysiology Laboratory, University of Ottawa Heart Institute, Ottawa, ON K1Y 4W7, Canada; 2 Department of Anesthesiology, Zhujiang Hospital, Southern Medical University, Guangzhou 510280, China; 3 Department of Cellular and Molecular Medicine, University of Ottawa, Ottawa, ON K1H 8M5, Canada

**Keywords:** Wnt signaling, cardiac sodium channel, Scn5a, glucose-rich medium

## Abstract

Wnt/β-catenin signaling has been shown to regulate gene expressions in cardiomyocytes. However, it is not known if this effect is dependent on the sex of cells or the glucose level in the culture medium. In the present study, ventricular myocytes were prepared from male and female neonatal rats and maintained in either a glucose-rich (25 mM) medium or a low-glucose (3 mM), lipid-rich medium. Real-time quantitative PCR was used to measure changes in target genes (*Axin2*, *Scn5a,* and *Tbx3*) after treatment with 1, 3, or 10 µM of CHIR-99021, an activator of Wnt/β-catenin signaling. CHIR induced similar changes in *Axin2, Tbx3, and Scn5a* transcripts in male and female NRVMs in both media, suggesting the absence of sex difference. However, cells in a high-glucose medium showed greater increases in *Axin2* and *Tbx3* transcripts than cells in a low-glucose medium. In addition, a low concentration of CHIR (1 µM) reduced the *Scn5a* transcript in cells in a high-glucose medium but not in a low-glucose medium, suggesting an increased sensitivity to Wnt signaling by high glucose. A non-linear relationship was identified between *Axin2* transcript upregulation and *Scn5a* transcript downregulation in CIHR-treated NRVMs. These data suggest that high glucose sensitizes both male and female cardiomyocytes to Wnt/β-catenin signaling.

## 1. Introduction

Wnt (Wingless and Int-1) ligands are proteins that are secreted from various types of source cells, and, upon binding to their receptors on target cells, they activate multiple intracellular pathways [1,2,3]. The canonical Wnt/β-catenin signaling pathway is evolutionally conserved and regulates the expression of target genes [2,3]. Activation of the receptor leads to inhibition of the glycogen synthase kinase-3β (GSK-3β) complex, a mediator of β-catenin degradation; β-catenin accumulates in the cytosol and then translocates into the nucleus, where it interacts with the T cell factor/lymphoid enhancer factor (TCF/LEF) family transcription factors regulating target gene transcription. While active during embryonic heart development, Wnt/β-catenin signaling maintains a low activity in healthy postnatal heart tissues. However, increased activity of the Wnt/β-catenin pathway has been found after cardiac injury and plays a role in arrhythmogenic heart disease, such as myocardial infarction and heart failure [4,5,6].

The *Scn5a* gene encodes the voltage-gated sodium channel α subunit (Na_v_1.5) in the heart. The Na_v_1.5-mediated Na^+^ current underlies the rapid upstroke of the action potentials in cardiomyocytes, triggering the contraction of the heart [7]. Reductions in the Na^+^ current are found in myocardial infarction [8] and Type 1 Brugada syndrome [9,10], promoting lethal ventricular tachyarrhythmias by reducing the action potential conduction velocity in myocardial tissues. Recent studies by us and other groups have demonstrated that Wnt/β-catenin signaling reduces the Na^+^ current by repressing the *Scn5a* gene in neonatal rat ventricular myocytes (NRVMs) [11,12], immortalized mouse atrial cells (HL-1 cells) [13,14], and human-induced pluripotent stem cell (iPSC)-derived cardiomyocytes [15]. In addition, reduced Na_v_1.5 has been found in adult ventricular tissues after Wnt/β-catenin signaling activation [12,16,17]. Reductions in the *Scn5a* transcript in post-MI mouse hearts were attenuated by cardiomyocyte-specific β-catenin deletion [18], suggesting a role of Wnt/β-catenin signaling in mediating the post-MI Na^+^ channel downregulation. The observation that *Scn5a*/Na_v_1.5 expression is reduced by Wnt/β-catenin signaling in both male and female iPSC-derived cardiomyocytes [15] suggests that this is a fundamental mechanism present in both sexes. However, it remains unknown if male and female cardiomyocytes respond to Wnt/β-catenin signaling activation in the same manner in terms of sensitivity or maximal effects.

Diabetes and hyperglycemia have been identified as risk factors for cardiovascular diseases, including cardiac arrhythmias [19,20,21]. Alterations in cardiac ion channels and excitability, which are proarrhythmic, have been shown in diabetic rabbit hearts and in guinea pig cardiomyocytes exposed to oscillations in glucose metabolism [22,23]. In addition, it has been shown that high glucose enhances the Wnt/β-catenin pathway in multiple cancer cell lines [24,25]. Because Wnt/β-catenin signaling regulates cardiac ion channels and promotes cardiac arrhythmias [18], it is important to answer the question whether glucose levels affect the sensitivity of cardiomyocytes to activators of the Wnt/β-catenin pathway as a potential contributing factor for increased cardiac arrhythmias in diabetics.

In the present study, neonatal rats of the same litters were separated into male and female groups before cardiomyocyte isolation and primary culture. This allowed the direct comparison of male and female cells for their responses to Wnt/β-catenin signaling. In addition, male and female NRMs were cultured in either a high-glucose medium or a low-glucose, lipid-rich medium [26], and their responses to CHIR-induced Wnt/β-catenin activation were compared. Our data showed that the CHIR-induced activation of Wnt/β-catenin signaling is independent of the sex of cardiomyocytes but is enhanced in a high-glucose medium. These findings, if confirmed in adult human cardiomyocytes, may provide a novel mechanism for diabetic heart disease [27].

## 2. Materials and Methods

### 2.1. Preparation of Male and Female Neonatal Rat Ventricular Myocytes

Two-day-old neonatal rats (Sprague Dawley, Harlan, Charles River, Montreal) were separated into male and female groups by their physical feature as described [28]. A total of 4 litters (8–12 neonatal rats in each litter) were used in this study. Neonatal rat ventricular myocytes (NRVMs) were isolated and cultured from male and female rats as we previously described [11,12]. The lower 1/3rd of the cardiac ventricular tissues were collected and minced into small pieces, which were then digested with 0.25% (*w*/*v*) trypsin (catalogue No. 15090046, Thermo Fisher Scientific, Waltham, MA, USA) in a calcium-free and magnesium-free Hanks’ Balanced Salt Solution (HBSS, catalogue No. 14175103, Thermo Fisher Scientific, Waltham, MA, USA) at 4 °C for overnight. The tissues were then subjected to digestion with 1 mg/mL of collagenase (type II, catalogue No. LS004176, Worthington Biochemical, Lakewood, NJ, USA) in the HBSS at 37 °C with gentle agitation. The released NRVMs were collected and resuspended in Medium 199 (catalogue No. 12340030, Thermo Fisher Scientific, Waltham, MA, USA), supplemented with 10% fetal bovine serum (FBS), 19.4 mM of glucose (final glucose = 25 mM), 2 mM of L-glutamine, 2 unit/mL of penicillin, 0.8 μg/mL of vitamin B12, 10 mM of HEPES, and 1× MEM of nonessential amino acids, which was designated as the Standard Medium. The cells were plated in uncoated flasks for 60 min, and attached cells (mostly cardiac fibroblasts) were removed by transferring the medium containing unattached cells to a new flask. After another 60 min incubation, the medium containing unattached cells (primarily cardiomyocytes) were counted and plated at 200,000 cells/cm^2^ in 6-well plates (catalogue No. 353046, Corning Inc., Corning, NY, USA) precoated with 0.1% gelatin (catalogue No. 07903, StemCell Technologies, Vancouver, BC, Canada). Cells of the high-glucose medium group were kept in the same medium (25 mM of glucose) described above, but the FBS was reduced to 2% at day 2 after cell isolation (designated as day 0).

### 2.2. Lipid-Rich Medium

A lipid-rich, low-glucose medium, which has been shown to maintain iPSC-derived cardiomyocytes and promote their metabolic maturation, was prepared by combining a glucose-free DMEM (catalogue No. 11966025, Thermo Fisher Scientific, Waltham, MA, USA), 3 mM of glucose, 10 mM of L-lactic acid, 5 μg/mL of Vitamin B12, 0.82 μM of biotin, 5 mM of creatine monohydrate, 2 mM of taurine, 2 mM of L-carnitine, 0.5 mM of ascorbic acid, 1× MEM of nonessential amino acids (catalogue No. 11140, Thermo Fisher Scientific, Waltham, MA, USA), 0.5% (*w*/*v*) Albumax (catalogue No. 11020021, Thermo Fisher Scientific, Waltham, MA, USA), 1× B-27 supplement (catalogue No. 17504044, Thermo Fisher Scientific, Waltham, MA, USA), and 1% knockout serum replacement (catalogue No. 10828028, Thermo Fisher Scientific, Waltham, MA, USA). Cells of the lipid-rich medium group were kept in this medium starting day 2 after cell isolation for 2 weeks with a medium change every 3 days before treatment for Wnt signaling activation.

### 2.3. Activation of Wnt/β-Catenin Signaling in NRVMs

NRVMs were treated with CHIR-99021 for the activation of Wnt/β-catenin signaling [11,12]. CHIR-99021 (catalogue No. S1263, Selleck Chemicals, Houston, TX, USA) was prepared in cell-culture grade DMSO (catalogue No. D2650, Sigma-Aldrich, St. Louis, MO, USA) at 100 mM as a stock solution. NRVMs were treated with CHIR-99021 at 1, 3, or 10 μM for 48 h before RNA extraction and qPCR analysis as described below. Control cells (0 CHIR) were treated with an equal volume of DMSO.

### 2.4. RNA Extraction and Real-Time Quantitative PCR

After treatment, NRVMs were collected in an RNAprotect Cell Reagent (catalogue No. 76526, Qiagen, Hilden, Germany), and the total RNA was isolated with an RNeasy mini kit (catalogue No. 74104, Qiagen, Hilden, Germany). Genomic DNA was removed by on-column digestion with RNase-Free DNase (catalogue No. 79254, Qiagen, Hilden, Germany). The same amount of total RNA (0.5 or 1.0 µg) from the samples was used for cDNA synthesis with a High Capacity cDNA Reverse Transcription Kit (catalogue No. 4368814, Thermo Fisher Scientific, Waltham, MA, USA). A real-time quantitative PCR was performed with a CFX Connect Real-Time PCR Detection System (Bio-Rad Laboratories, Hercules, CA, USA) using the iTaq Universal SYBR Green Supermix (catalogue No. 1725121, Bio-Rad Laboratories, Hercules, CA, USA). qPCR primer information was included in Table 1. Levels of target gene transcripts were normalized to a validated cardiomyocyte housekeeping gene (*Hprt1* [11,12]) in the same samples. Results were analyzed with the 2−ΔΔ C(t) method.

### 2.5. Statistical Analysis

Data are expressed as the mean ± standard error of mean (SEM) with *p* < 0.05 considered significant. Individual data points are included in the summary figures. Statistical analyses were conducted using GraphPad Prism (Version 10.3.0). Differences between the two means were evaluated by a two-tailed Student’s *t*-test. Differences among multiple means were assessed by a one-way or two-way analysis of variance (ANOVA). When significance was detected by the ANOVA, differences among individual means were evaluated post hoc by the Sidák or Bonferroni test as indicated in the figure legends.

## 3. Results

### 3.1. Preparation of Male and Female Neonatal Rat Ventricular Myocytes (NRVMs)

**Figure 1:**
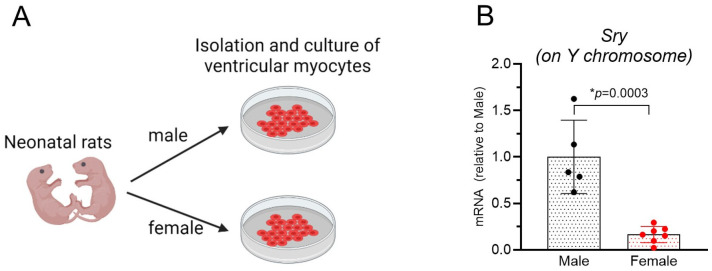
Preparation of male and female neonatal rat ventricular myocytes: (**A**). Male and female 2-day-old rats were separated by their genitalia feature (a greater anogenital distance in males) (Created with BioRender.com with authorization). (**B**). Successful separation was confirmed by a greater level of *Sry* mRNA (a Y-chromosome gene) in male cells, * *p* = 0.0003 by a two-tailed Student’s *t*-test, *n* = 5–7.

In our previous studies, male and female neonatal rats were mixed for NRVM isolation [11,12]. Therefore, it is unclear if male and female NRVMs respond to Wnt/β-catenin signaling differently. To address this issue, we separated male and female neonatal rats before NRVM isolation (Figure 1A) based on their anatomical feature in the perineum region, i.e., a greater anogenital distance in males, as described by McCarthy M. [28]. RT-qPCR analysis showed a greater (*p* = 0.0003) level of Sry mRNA, a Y-chromosome gene, in male than in female NRVMs (Figure 1B), demonstrating the successful separation of male and female cells.

### 3.2. Baseline Gene Expressions in Male and Female NRVMs in Glucose-Rich and Lipid-Rich Media

**Figure 2:**
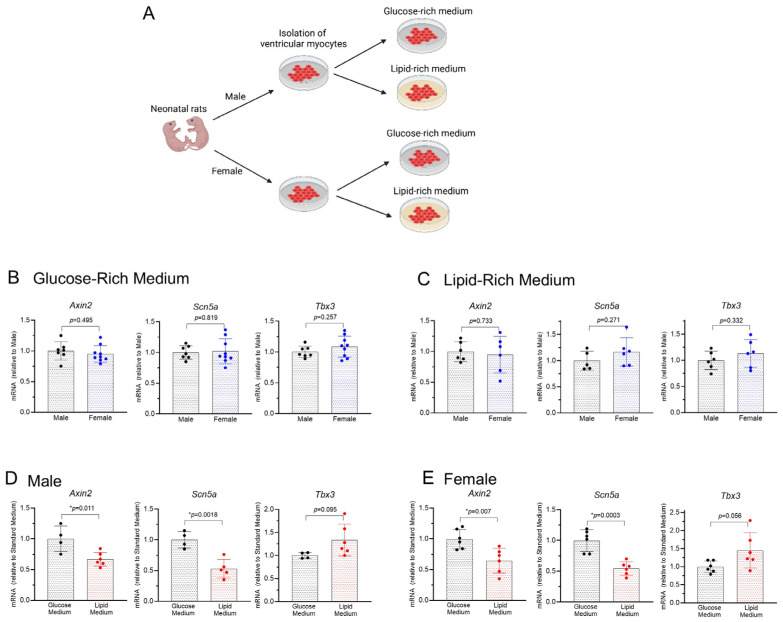
Baseline transcript levels of *Axin2*, *Scn5a, and Tbx3* in male and female NRVMs cultured in either aglucose-rich medium or alipid-rich medium: (**A**). Diagram of four groups of cells with different sexes and culture media (Created with BioRender.com with authorization). (**B**). Gene expressions in male and female cells cultured in a glucose-rich medium. (**C**). Gene expressions in male and female cells cultured in a lipid-rich medium. (**D**). Comparison of male cells cultured in glucose-rich and lipid-rich media. (**E**). Comparison of female cells cultured in glucose-rich and lipid-rich media. Note: panels (**B**,**C**) and panels (**D**,**E**) are the same data set presented in different formats for clearer comparison. * *p* < 0.05. *p* values are indicated for each figure, and data were analyzed by a two-tailed Student’s *t*-test, *n* = 4–6.

After the preparation of male and female NRVMs, cells were cultured in either the glucose-rich medium or the lipid-rich medium (Figure 2A). RT-qPCR showed that the transcripts of *Axin2*, *Scn5a,* and *Tbx3,* three genes known to be regulated by Wnt/β-catenin signaling in cardiomyocytes [11,12], are not different (*p* > 0.257) between male and female NRVMs regardless of the cell culture media (Figure 2B,C). However, when comparing the cells in the two different media, higher levels of *Axin2* and *Scn5a* transcripts were found in the glucose-rich medium group regardless of the sex of the cells (Figure 2D,E). These data suggest that the baseline expression levels of *Axin2* and *Scn5a* are not dependent on the sex of the cells but are increased when a high level of glucose is present as the metabolic substrate.

### 3.3. Axin2 mRNA Upregulation Is Sex-Independent, but Is Greater in Glucose-Rich Medium

**Figure 3:**
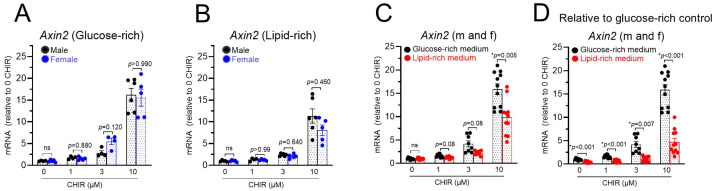
The upregulation of *Axin2* by CHIR-induced Wnt signaling activation is not dependent on the sex of cells but is greater in a glucose-rich medium. (**A**). The upregulation of *Axin2* mRNA by CHIR treatment in a glucose-rich medium. (**B**). The upregulation of *Axin2* mRNA by CHIR treatment in a lipid-rich medium. (**C**). Data from A and B were combined to show greater *Axin2* mRNA increases in a glucose-rich medium. (**D**). Data in (**C**) were normalized to the glucose-rich control (0 CHIR) to show greater *Axin2* mRNA in a glucose-rich medium at all the different concentrations of CHIR. ns = not significant. * *p* < 0.05. *p* values are indicated, and data were analyzed by two-way ANOVA with a Sidák test, *n* = 4–11.

Axin2 gene transcription is directly regulated by Wnt/β-catenin signaling [29], and Axin2 mRNA increases have been widely used as an index of Wnt/β-catenin pathway activation in different cell types, including cardiomyocytes [11,12,15]. The treatment of NRVMs with different concentrations (1, 3, and 10 µM) of CHIR-99021, which is an inhibitor of GSK-3β, led to dose-dependent increases in Axin2 mRNA. Similar levels of Axin2 mRNA increases were observed in both male and female cells regardless of the cell culture media (Figure 3A,B), suggesting that the effect was not dependent on the sex of the cells. Therefore, data from male and female cells were combined for the analysis of effects of cell culture media. CHIR-induced Axin2 mRNA increases were higher (*p* < 0.001) in glucose-rich media (15.9 ± 1.2 increases by 10 µM of CHIR, *n* = 11) than in the lipid-rich medium (9.9 ± 1.1 increases by 10 µM of CHIR, *n* = 11) (Figure 3C). When all the data were normalized to the glucose-rich medium control group (0 CHIR), lower levels of Axin2 mRNA were seen in the lipid-rich group at all the different concentrations of CHIR (Figure 3D).

### 3.4. Scn5a mRNA Downregulation Is Sex-Independent, but Is More Sensitive to CHIR in Glucose-Rich Medium

**Figure 4:**
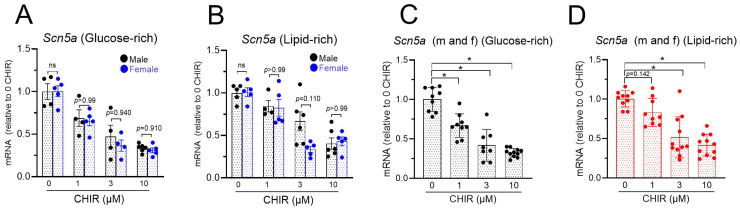
Downregulation of *Scn5a* by CHIR-induced Wnt signaling activation is not dependent on sex of cells but is more sensitive in glucose-rich medium: (**A**). Downregulation of *Scn5a* mRNA by CHIR treatment in glucose-rich medium. (**B**). Downregulation of *Scn5a* mRNA by CHIR treatment in lipid-rich medium. (**C**). Male and female data from panel (**A**) were combined to show reductions in Scn5a mRNA in glucose-rich medium. (**D**). Male and female data in panel (**B**) were combined to show reductions in Scn5a mRNA in lipid-rich medium. ns = not significant. * *p* < 0.01 and data were analyzed by two-way ANOVA with Sidák test or one-way ANOVA with Bonferroni test, *n* = 4–11.

Scn5a is a gene that we have previously shown to be downregulated by Wnt/β-catenin signaling in mixed male and female NRVMs [11]. In the present study, similar levels of Scn5a mRNA reductions were observed in both male and female cells regardless of the cell culture media (Figure 4A,B), suggesting that the effect was not dependent on the sex of the cells. When male and female cell data were combined, a higher sensitivity of Scn5a mRNA to CHIR treatment was identified for the glucose-rich group: Scn5a mRNA was reduced by the lowest concentration (1 µM) of CHIR in the glucose-rich medium group (*p* < 0.01) but not in the lipid-rich medium group (*p* = 0.142) (Figure 4C,D). However, the maximum reductions in Scn5a mRNA at the highest concentration (10 µM) of CHIR were not statistically different (*p* = 0.220) between the glucose-rich group (0.32 ± 0.02, *n* = 11) and the lipid-rich group (0.42 ± 0.04, *n* = 11).

### 3.5. Tbx3 mRNA Upregulation Is Sex-Independent, but Is Greater in Glucose-Rich Medium

**Figure 5:**
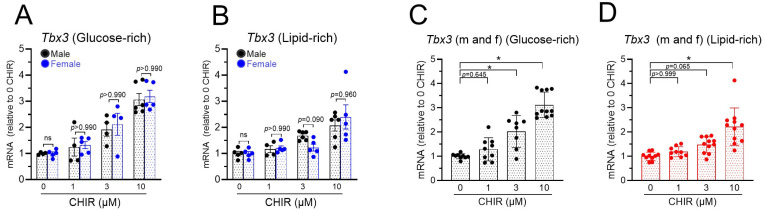
Upregulation of *Tbx3* by CHIR-induced Wnt signaling activation is not dependent on sex of cells, but is greater in glucose-rich medium: (**A**). Upregulation of *Tbx3* mRNA by CHIR treatment in glucose-rich medium. (**B**). Upregulation of *Tbx3* mRNA by CHIR treatment in lipid-rich medium. (**C**). Male and female data from panel (**A**) were combined to show increases in Tbx3 mRNA in glucose-rich medium. (**D**). Male and female data in panel (**B**) were combined to show increases in Tbx3 mRNA in lipid-rich medium. ns = not significant. * *p* < 0.01 and data were analyzed by two-way ANOVA with Sidák test or one-way ANOVA with Bonferroni test, *n* = 4–11.

Our previous studies have demonstrated that Tbx3 mRNA is increased in mixed male and female NRVMs [12]. In the present study, similar levels of Tbx3 mRNA upregulations were found in both male and female cells regardless of the cell culture media (Figure 5A,B), suggesting that the effect was independent of the sex of the cells. Analysis with male and female data combined showed that the lowest concentration of CHIR that led to Tbx3 mRNA increases was 3 µM in the glucose-rich group but 10 µM in the lipid-rich group (Figure 5C,D). In addition, 10 µM of CHIR led to greater (*p* = 0.02) increases in Tbx3 mRNA in the glucose-rich group (3.12 ± 0.16, *n* = 11) than in the lipid-rich group (2.22 ± 0.23, *n* = 11).

### 3.6. Non-Linear Correlation Between Axin2 mRNA Increases and Scn5a mRNA Reductions

**Figure 6:**
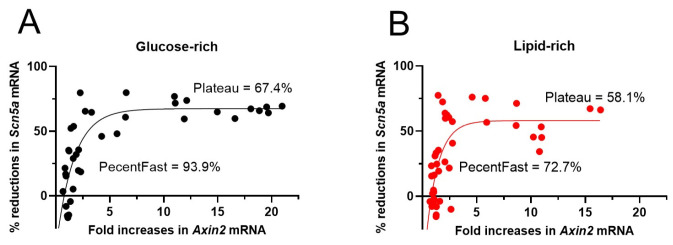
The association of percent (%) reductions in *Scn5a* mRNA and fold increases in *Axin2* mRNA in NRVMs of both sexes treated with different concentrations of CHIR. (**A**,**B**) Curves were fitted with the Two-Phase Association Exponential Regression using GraphPad Prism program (version 10.3.0). Model: Y = Y_0_ + SpanFast*(1−exp(−KFast*X)) + SpanSlow*(1−exp(−KSlow*X)), in which SpanFast = (Plateau−Y_0_)*PercentFast*0.01 and SpanSlow = (Plateau-Y_0_)*(100−PercentFast)*0.01). Plateaus are the Y values (i.e., percent reductions in *Scn5a* mRNA) at infinite times.

Increases in *Axin2* mRNA have been used as an index of the degree of Wnt/β-catenin pathway activation [15]. Fitting the changes in *Axin2* and *Scn5a* mRNA in CHIR-treated NRVMs showed a non-linear relationship: two-phase association fitting showed a fast phase and a slow phase with 93.9% and 72.7% of the *Scn5a* mRNA reductions completed in the fast phase in the glucose-rich group and lipid-rich group, respectively (Figure 6). In addition, the maximum reduction in *Scn5a* mRNA (plateau) is also higher in the glucose-rich group (67.4%) than in the lipid-rich group (58.1%) (Figure 6). These observations suggest that NRVMs maintained in the glucose-rich medium have a greater sensitivity to Wnt/β-catenin activation. These observations also suggest that changes in *Scn5a* mRNA are a more sensitive index than changes in *Axin2* mRNA for CHIR-induced Wnt/β-catenin pathway activation in cultured NRVMs.

### 3.7. Non-Linear Correlation Between Axin2 and Tbx3 mRNA Upregulations

**Figure 7:**
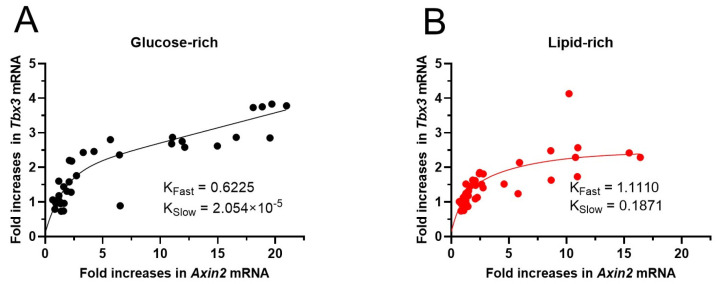
The association of fold increases in *Tbx3* and *Axin2* mRNA in NRVMs of both sexes treated with different concentrations of CHIR. (**A**,**B**) Curves were fitted with the Two-Phase Association Exponential Regression using GraphPad Prism program (version 10.3.0). Model: Y = Y_0_ + SpanFast*(1−exp(−K_Fast_*X)) + SpanSlow*(1−exp(−K_Slow_*X)), in which K_Fast_ and K_Slow_ are the fast and slow time constants, respectively.

Similarly, the increases in *Axin2* and *Tbx3* mRNA in CHIR-treated NRVMs also showed a non-linear relationship: two-phase association fitting showed a fast phase and a slow phase in both glucose-rich and lipid-rich groups (Figure 7). The smaller time constants in the glucose-rich group (K_Fast_ = 0.6225, K_Slow_ = 2.054 × 10^−5^) than in the lipid-rich group (K_Fast_ = 1.1110, K_Slow_ = 0.1871) suggest a greater sensitivity to Wnt/β-catenin activation in cells maintained in the glucose-rich medium.

### 3.8. Linear Correlation Between Tbx3 mRNA Increases and Scn5a mRNA Reductions

**Figure 8:**
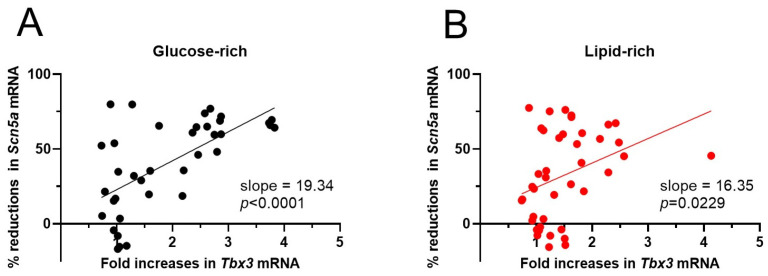
The association of *Tbx3* mRNA increases and *Scn5a* mRNA reductions in NRVMs of both sexes treated with different concentrations of CHIR. (**A**,**B**) Curves were fitted with Simple Linear Regression using GraphPad Prism program (version 10.3.0).

Fitting the *Tbx3* mRNA increases and *Scn5a* mRNA reductions showed a linear relationship (Figure 8). The higher slope in the glucose-rich group (19.34) than in the lipid-rich group (16.35) suggests greater reductions in *Scn5a* mRNA at the same level of the *Tbx3* mRNA increase during CHIR-induced Wnt/β-catenin activation in cells maintained in the glucose-rich medium.

## 4. Discussion

The biological sex has been shown to regulate both cardiac physiology and disease [30]. Although the roles of sex hormones in sex differences have been demonstrated, the importance of non-hormone mechanisms such as sex chromosomes, as a result of the expression of Y chromosome genes in males and the differential X chromosome gene expressions between males and females [31,32,33], has been recognized. The present study included male and female cardiomyocytes prepared from the same litters of neonatal rats, allowing the direct comparison of their responses to Wnt/β-catenin pathway activation. The similar baseline levels of *Axin2*, *Scn5a*, and *Tbx3* transcripts between male and female NRVMs suggest the absence of sex difference. CHIR-99021 is a small molecule that permeates the plasma membrane and inhibits GSK-3β in the cytosol, leading to activation of the Wnt/β-catenin pathway [11]. The same levels of *Axin2* and *Tbx3* transcript upregulation and *Scn5a* transcript downregulation in male and female NRVMs after CHIR-99021 treatments suggest that the regulation of these Wnt-responsive genes by direct GSK-3β inhibition is not dependent on the sex of the cardiomyocytes. These observations are consistent with the role of the Wnt/β-catenin pathway as a fundamental signaling pathway that is highly conserved and regulates early embryonic development. However, future studies are needed to investigate if sex hormones regulate the Wnt/β-catenin pathway activation in cardiomyocytes and if Wnt protein-induced Wnt/β-catenin pathway activation via plasma member receptors [34] is dependent on the sex of the cardiomyocytes.

Because it is challenging to maintain primary cardiomyocytes in culture, a glucose-rich medium is frequently used for the culture of neonatal rat cardiomyocytes. Recent studies have shown that a low-glucose, lipid-rich medium can be used to replace the glucose-rich medium for the culture of iPSC-derived cardiomyocytes [26,35]. In addition, because the lipid-rich medium provides more physiological substrates for cardiomyocyte metabolism, it has been shown to promote the metabolic maturation of iPSC-derived cardiomyocytes that typically show an immature phenotype [26]. The present study demonstrated that the regulations of *Axin2*, *Scn5a,* and *Tbx3* transcripts by CHIR-induced Wnt pathway activation are found in NRVMs cultured either in the glucose-rich medium or the lipid-rich medium. However, the degree of Wnt/β-catenin pathway activation, as gauged by *Axin2* mRNA upregulation, as well as the changes in Wnt target genes *Scn5a* and *Tbx3* mRNA, is greater in NRVMs in a glucose-rich (25 mM) medium than in the lipid-rich (3 mM of glucose) medium despite identical CHIR treatments. This suggests that high glucose sensitizes cardiomyocytes to CHIR-induced Wnt pathway activation. In cancer cell lines, glucose has been shown to enhance β-catenin acetylation at K354, which facilitates its nuclear retention for prolonged effects on target gene transcription during Wnt/β-catenin pathway activation [24]. Another study showed that a high-glucose medium activates the Wnt/β-catenin pathway in hepatocellular carcinoma by reducing the expression of DKK4, a Wnt pathway inhibitor [25]. Future studies are warranted to investigate if the regulation of Wnt pathways by glucose in cardiomyocytes is mediated by the same mechanisms found in cancer cells or by different mechanisms and to investigate if sex hormones or metabolic factors regulate the activation of Wnt/β-catenin signaling and its role in heart disease pathogenesis in preclinical models of diabetes.

A limitation of the present study is that, in addition to the different glucose levels, the lipid-rich medium also contained additional supplements, which were included primarily to facilitate fatty acid transportation and oxidation in cardiomyocytes. Future studies are needed to test if any of these additional supplements also contribute to the lower sensitivity to Wnt pathway activators in cells of the low-glucose medium. In addition, our recent studies using Brugada syndrome iPSC-derived cardiomyocytes showed that Na_v_1.5, the Na^+^ channel protein encoded by *Scn5a*, is regulated by Wnt/β-catenin signaling at both the transcriptional and post-transcriptional levels [15]. The present study investigated alterations in the *Scn5a* transcript after CHIR treatments, and future studies are needed to investigate if alterations in the Na_v_1.5 protein via the post-transcriptional mechanism are also regulated by glucose levels.

## 5. Conclusions

Our data demonstrated that CHIR-induced activation of Wnt/β-catenin signaling is independent of the sex of cardiomyocytes but is enhanced when cells are maintained in a high-glucose medium. Because hyperglycemia is a hallmark of diabetes, and Wnt/β-catenin signaling is known to play a role in the pathogenesis of heart disease, our findings, if confirmed in adult human cardiomyocytes, may provide a new mechanism for diabetic heart disease.

## Figures and Tables

**Table 1:** Primers for SYBR green qPCR.

Genes	Forward (5′ to 3′)	Reverse (5′ to 3′)	Amplicon Size (bp)	Target Exon (s)
*Axin2*	TCCTTACCGCATGGGGAGTA	GTGGGTTCTCGGGAAGTGAG	100	3–4
*Scn5a*	TATGTTGAGTACACCTTCACTGC	GCCCAGGTCCACAAATTCAG	165	5–6
*Sry*	GCTGCAATGGGACAACAACC	TTCTTGGAGGACTGGTGTGC	78	1
*Tbx3*	AGACGTAGAAGACGACCCCA	AGGGAACATTCGCCTTCCTG	112	1–2
*Hprt1*	ACAGGCCAGACTTTGTTGGA	TGCCGCTGTCTTTTAGGCTT	149	7–8

## Data Availability

All original raw data from which graphical and/or tabular summary data were generated are archived and fully available to The Journal upon reasonable request.
